# Mediastinitis after endobronchial ultrasound with transbronchial needle aspiration resulting in postpneumonectomy empyema

**DOI:** 10.1016/j.xjtc.2024.03.012

**Published:** 2024-03-26

**Authors:** Nathaniel L. Robinson, Ryan D. Watkins, Luis F. Tapias

**Affiliations:** a Department of Surgery, Mayo Clinic, Rochester, Minn; b Division of Thoracic Surgery, Mayo Clinic, Rochester, Minn

**Figure:**
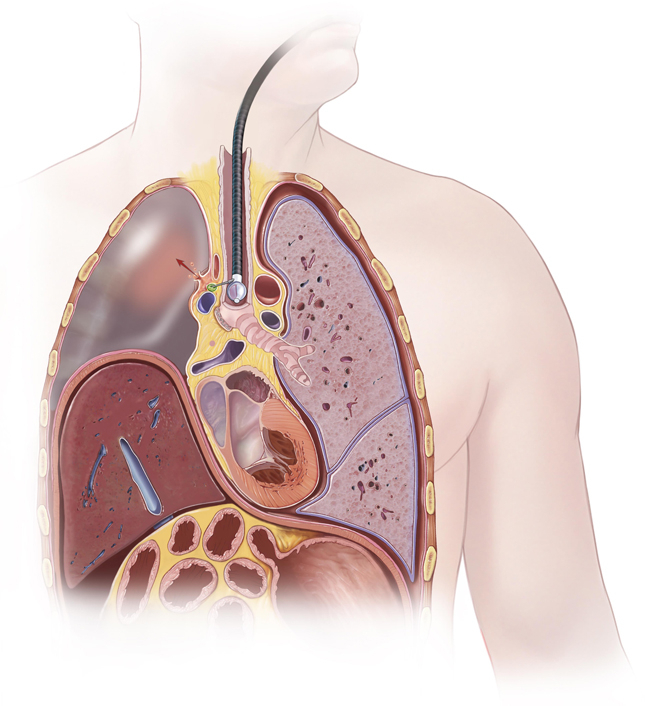
Illustrative diagram of clinical scenario depicting process of infection development.

**Central Message**

Endobronchial ultrasound–guided sampling of paratracheal lymph nodes seeded with bacteria led to a mediastinal infection with resultant abscess and progression to postpneumonectomy space empyema.

Endobronchial ultrasound with transbronchial needle aspiration (EBUS-TBNA) has been proven safe and effective for invasive mediastinal staging or sampling of mediastinal lesions.^1^ Infectious complications after EBUS-TBNA have been observed in 0.19% of procedures.^2^ Herein, we present a case of postpneumonectomy space empyema secondary to mediastinitis after EBUS-TBNA. The institutional review board or equivalent ethics committee of the Mayo Clinic did not approve this study, as it was deemed exempt. The subject provided informed written consent for the publication of the study data.

## Case Presentation

A 63-year-old male patient underwent right pneumonectomy and 4 cycles of adjuvant platinum doublet chemotherapy for stage IIIA (pT3N1M0) squamous cell carcinoma. Sixteen months later, surveillance imaging identified enlarged, fluorodeoxyglucose-avid, mediastinal lymph nodes in stations 2R and 7 concerning for recurrence. EBUS-TBNA was performed via a 22-gauge needle, and the lymph nodes in station 2R and 7 were noted to be 15 and 25 mm, respectively. Final pathology confirmed recurrence in both stations.

**Figure 1:**
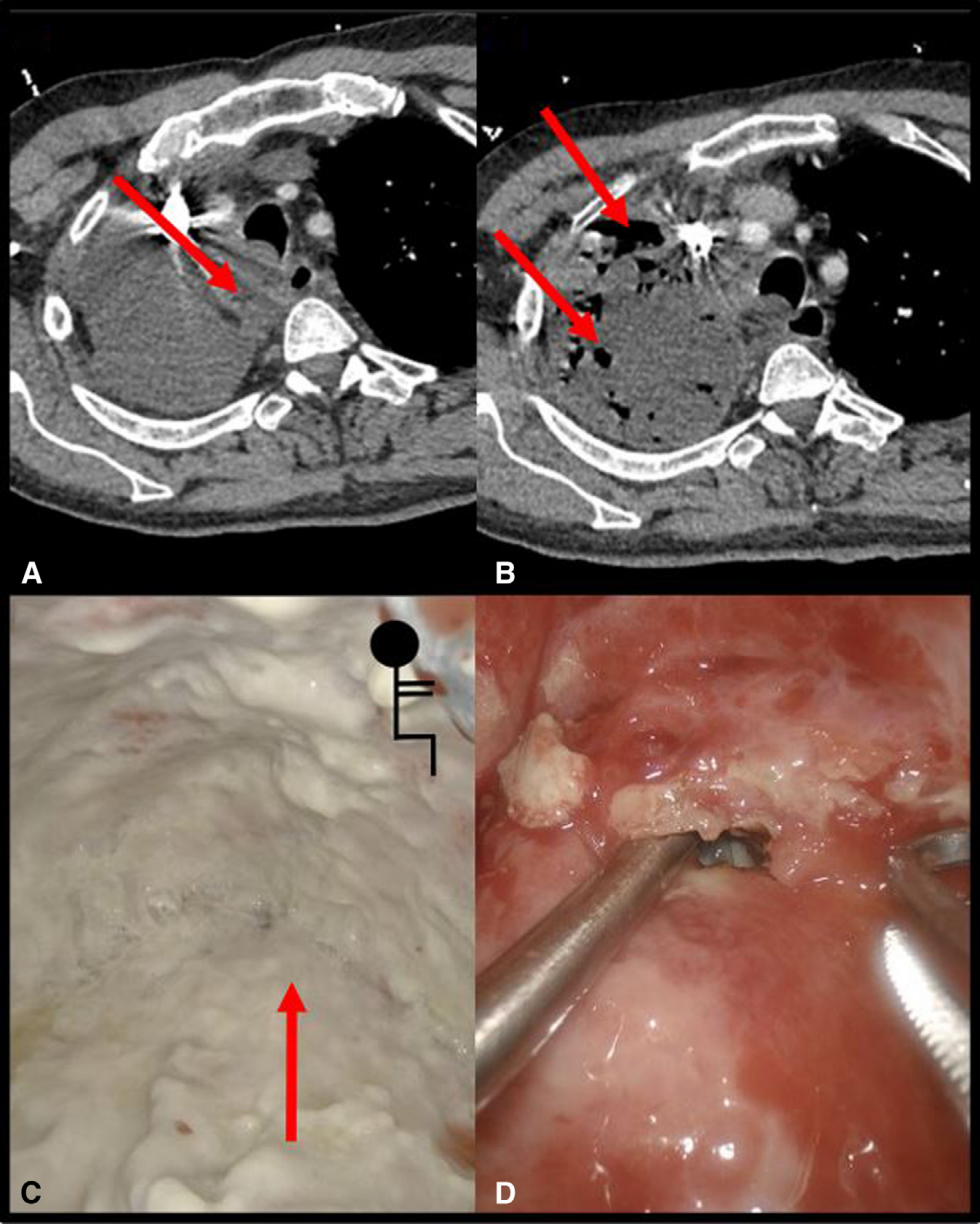
Radiographic and intraoperative clinical findings. A, Contrast-enhanced axial CT 10 days after the procedure identifying right paratracheal fluid collection. B, Contrast-enhanced axial CT 17 days after the procedure identifying progression of right paratracheal fluid collection and postpneumonectomy space gas. C, Intraoperative photograph of the right thoracic cavity with diffuse fibropurulent material throughout the chest. *Arrow* indicates the paratracheal mediastinal abscess before irrigation. D, Intraoperative photograph of the right paratracheal mediastinal abscess cavity in communication with the right pleural cavity. *CT*, Computed tomography.

Ten days later, he presented to the emergency department with atrial fibrillation, fevers, cough, and nausea. Computed tomography of the chest was read as normal during this presentation at an outside institution. In retrospective review, however, there was a small fluid collection in the right upper paratracheal space (Figure 1, *A*). One week after persistent symptoms, additional imaging identified gas in the right postpneumonectomy space and an air-fluid collection in the mediastinum (Figure 1, *B*). Thoracentesis confirmed empyema, and chest tube insertion drained 2 L of purulent fluid. Culture-directed antibiotic therapy and alteplase/dornase instillations were initiated before transfer to our institution. Of note, the patient was not immunosuppressed, was not taking steroids, and was not a smoker. The note from bronchoscopy and EBUS noted no abnormal secretions.

In the operating room, flexible bronchoscopy was performed to rule out a bronchopleural fistula. Video-assisted thoracoscopic surgery exploration revealed diffuse fibrinopurulent pleuritis atypically focused at the apex of the thoracic cavity (Figure 1, *C*). A right upper paratracheal mediastinal abscess with spontaneous drainage into the right postpneumonectomy space was discovered (Figure 1, *D*). This correlated with the radiographic mediastinal collection at the site of the biopsied station 2R lymph node. Debridement of the postpneumonectomy space was performed. A drain was placed in the mediastinal abscess, and pigtail drains were placed in the apex and base of the chest for planned postoperative irrigation of the postpneumonectomy space. Pleural fluid cultures identified myriad aerobic and anaerobic oral flora, including *Streptococcus anginosus*, *Haemophilus parainfluenzae*, *Fusobacterium nucleatum*, *Veillonella* species, *Parvimonas micra*, *Lancefieldella rimae*, *Segatella buccae*, *Streptococcus constellatus*, and *Schaalia turicensis*.

Postoperatively, the right postpneumonectomy space was irrigated with 400 mL of modified DABs (20 μg/mL gentamycin and polymyxin B 500 units/mL in 0.9% NaCl solution) twice daily for 10 days through the pigtail drains, allowing incubation and drainage. Pleural fluid cultures on postoperative day 5 and 10 were negative. The postpneumonectomy space was filled with modified DABs, and drains were removed on postoperative day 13. He was discharged with intravenous ceftriaxone and oral metronidazole to complete 4 weeks of therapy. After 7 months of follow-up, he has not experienced recurrence of empyema and is undergoing treatment for recurrent lung cancer with definitive chemoradiation to include 6000 cGy in 30 fractions total with concurrent carboplatin and paclitaxel.

## Discussion

**Figure 2:**
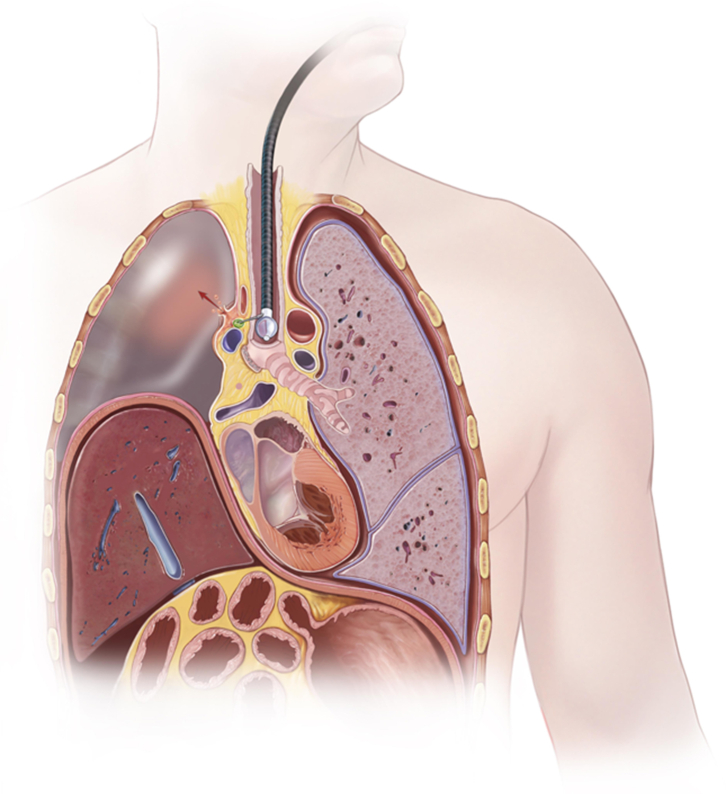
Diagram of clinical scenario. Schematic of endobronchial ultrasound-guided sampling of paratracheal lymph node with resultant abscess and progression to postpneumonectomy empyema.

Mediastinitis as a complication of EBUS is rare and reported at 0.1%, but consequences can be severe.^2^ In this case, the associated fluid-filled postpneumonectomy space presented an increased risk and became secondarily infected after a mediastinal abscess ruptured into the right side of the chest (Figure 2). Furthermore, it has been reported that approximately 7% of patients undergoing EBUS experience bacteremia with oropharyngeal flora,^3^ raising the possibility of potential hematogenous dissemination into a fluid-filled postpneumonectomy space. Although prophylactic antibiotics are not recommended routinely when performing EBUS-TBNA,^4^ prophylactic antibiotics should be considered when sampling ipsilateral mediastinal lymph nodes in such high-risk patients with a persistent fluid-filled postpneumonectomy space. In this report, a myriad of bacteria were present. A single dose of pre-EBUS-TBNA prophylactic antibiotics that cover oropharyngeal flora may not have covered every possible infectious organism, but in our opinion, is a reasonable consideration to prevent mediastinitis and abscess formation, given the implications of the potential complications as shown in the present case. Pleural debridement and irrigation of the postpneumonectomy space, resembling a modified Claggett procedure, has been described for early infections complicated by bronchopleural fistulae with good results.^5^ This also proved effective in our case.

## Conclusions

EBUS-associated infections in a patient after pneumonectomy can have profound consequences requiring surgical intervention. Infectious complications should be suspected in the setting of invasive mediastinal testing after pneumonectomy and treated early and aggressively. Prophylactic antibiotic administration for EBUS in high-risk situations, such as patients with fluid-filled postpneumonectomy space, should be considered.

## Conflict of Interest Statement

L.F.T. reported consultant for AtriCure Inc., AstraZeneca, and Intuitive Surgical. All other authors reported no conflicts of interest.

The *Journal* policy requires editors and reviewers to disclose conflicts of interest and to decline handling or reviewing manuscripts for which they may have a conflict of interest. The editors and reviewers of this article have no conflicts of interest.
